# Arterial Stiffness in Cancer Survivors: The Prognostic Value of Estimated Pulse Wave Velocity in US Cancer Population From the National Health and Nutrition Examination Survey 2009 to 2018

**DOI:** 10.1161/JAHA.125.041645

**Published:** 2025-09-19

**Authors:** Mustafa Al‐jarshawi, Akhmetzhan Galimzhanov, Marco Zimarino, Islam Y. Elgendy, Carlos Diaz‐Arocutipa, Ofer Kobo, Mamas A. Mamas

**Affiliations:** ^1^ Keele Cardiovascular Research Group, Centre for Prognosis Research Keele University Stoke‐on‐Trent UK; ^2^ Outpatient Clinic #7, Department of Internal Medicine Semey Kazakhstan; ^3^ Department of Cardiology SS.Annunziata Hospital—ASL and University “G. D’Annunzio” of Chieti Chieti Italy; ^4^ Division of Cardiovascular Medicine, Gill and Vascular Heart Institute University of Kentucky Lexington KY USA; ^5^ Unidad de Revisiones Sistemáticas y Meta‐análisis (URSIGET), Vicerrectorado de Investigación Universidad San Ignacio de Loyola Lima Peru

**Keywords:** arterial stiffness, cancer survivors, early vascular aging, estimated pulse wave velocity, death, Cardiovascular Disease, Epidemiology, Risk Factors, Primary Prevention, Mortality/Survival

## Abstract

**Background:**

Estimated pulse wave velocity (ePWV), a marker of arterial stiffness, is strongly associated with cardiovascular events and all‐cause death in the general population. This study evaluates the association of arterial stiffness, defined by the ePWV equation, with all‐cause and cardiovascular death and identifies an optimal ePWV threshold for risk stratification in cancer survivors.

**Methods:**

Using data from the National Health and Nutrition Examination Survey (2009–2018), we analyzed a cohort representing more than 105 million US cancer survivors. Multivariable Cox regression and Kaplan–Meier analyses assessed the association between ePWV and outcomes. Dose‐dependent relationships were explored, and an optimal ePWV threshold was determined through maximally selected rank statistics.

**Results:**

During a median follow‐up of 59 months, a total of 105 869 670 weighted cancer survivors (2234 unweighted) were analyzed, recording 435 all‐cause and 91 cardiovascular deaths. Early vascular aging (ePWV ≥9.4 m/s) was present in 54.6% of participants. Each 1 m/s increase in ePWV was associated with higher risks of all‐cause death (29%) and cardiovascular death (46%) (hazard ratio [HR], 1.29 [95% CI, 1.19–1.40] and 1.46 [95% CI, 1.20–1.76], respectively, both *P*<0.001). Those with ePWV ≥12.05 m/s demonstrated higher risks of all‐cause and cardiovascular death (HR, 1.70 [95% CI, 1.35–2.13] and 2.53 [95% CI, 1.56–4.12], respectively, both *P*<0.001).

**Conclusions:**

Arterial stiffness, assessed by ePWV, demonstrates prognostic utility in cancer survivors, showing a nonlinear association with all‐cause and cardiovascular death, independent of demographic, socioeconomic, traditional cardiovascular risk factors not included in the ePWV equation, and baseline cardiovascular disease.

**Nonstandard Abbreviations and Acronyms**
cfPWV: carotid–femoral pulse wave velocity
ePWV: estimated pulse wave velocity
EVA: early vascular aging
MBP: mean blood pressure
MORGAM: Monica Risk, Genetics, Archiving and Monograph
NHANES: National Health and Nutrition Examination Survey
PWV: pulse wave velocity
SPRINT: Systolic Blood Pressure Intervention Trial

**Clinical Perspective**

**What Is New?**

- Arterial stiffness, estimated using the noninvasive estimated pulse wave velocity equation, is associated with increased all‐cause and cardiovascular death in US cancer survivors.
- Early vascular aging, defined as estimated pulse wave velocity ≥9.4 m/s, was present in more than half of the cohort, with the highest prevalence observed in rectal, prostate, esophageal, and bladder cancer survivors.

**What Are the Clinical Implications?**

- Estimated pulse wave velocity provides a practical, noninvasive estimate of arterial stiffness that may help identify cancer survivors at increased risk of early vascular aging and adverse mortality outcomes.

Hypertension is the leading cardiovascular risk factor for the future development of cardiovascular disease (CVD),^1^ contributing to 13.5% of all deaths annually worldwide. Cancer survivors face an increased risk of developing high blood pressure compared with individuals without cancer, primarily due to treatment‐related vascular toxicity, radiotherapy, and shared cardiovascular risk factors.^2^, ^3^ In 2024, the incidence of hypertension among cancer survivors was reported to be 364.6 per 10 000 person‐years compared with 211 per 10 000 person‐years in the general population.^4^, ^5^

High blood pressure plays a key role in arterial stiffness and contributes to vascular aging through mechanisms such as endothelial dysfunction, collagen deposition, and reduced arterial elasticity.^6^ Early vascular aging (EVA), first described in 2008, describes the premature onset of these structural and functional alterations in the arteries, resembling the physiological aging process but occurring at an earlier stage.^7^

The most common clinical method for detecting arterial stiffness is measuring pulse wave velocity (PWV).^8^ PWV quantifies the speed at which a pressure wave travels along the arterial wall after each cardiac contraction. The carotid–femoral PWV (cfPWV) is considered the gold standard for evaluating arterial stiffness and vascular age.^9^, ^10^ Studies have consistently demonstrated that arterial stiffness, assessed through PWV, is strongly associated with long‐term health outcomes, including CVD events and all‐cause death.^11^, ^12^, ^13^, ^14^ Professional society guidelines on the management of elevated blood pressure and hypertension also recommend the measurement of cfPWV to improve CVD risk stratification beyond traditional risk factors.^15^, ^16^ However, the routine use of cfPWV in clinical practice has been limited by its reliance on specialized equipment and specific technical expertise.^17^, ^18^, ^19^

The estimated PWV (ePWV) has been proposed as an alternative measure to cfPWV.^20^ Derived from age and blood pressure, ePWV is a noninvasive index of arterial stiffness^6^ that has shown a strong correlation with cfPWV values.^21^, ^22^ In 2019, a secondary analysis of data from SPRINT (Systolic Blood Pressure Intervention Trial) demonstrated that arterial stiffness estimated by ePWV has a predictive role for CVD events, including myocardial infarction, acute coronary syndromes, stroke, heart failure, or death from cardiovascular causes, and all‐cause death, independent of the traditional cardiovascular risk scores, which consider age and blood pressure separately rather than combining them into a vascular measure as ePWV does.^20^ Subsequent studies have further investigated the prognostic value of ePWV in different patient groups, including individuals with hypertension, diabetes, and chronic kidney disease, and critically ill patients with coronary heart disease, stroke, and stable angina undergoing elective coronary angiography, demonstrating its utility as a predictor of CVD events and all‐cause death in these populations.^18^, ^23^, ^24^, ^25^, ^26^, ^27^, ^28^, ^29^ However, whether ePWV, as a noninvasive marker of arterial stiffness, retains similar prognostic value in cancer survivors remains unclear. This population is uniquely affected by treatment‐induced vascular toxicity, systemic inflammation, endothelial dysfunction, and accelerated vascular aging.^30^ As such, arterial stiffness estimated by the ePWV equation may amplify mortality risk in this group, highlighting the need for population‐specific analysis.

This study aims to investigate the association of arterial stiffness, as defined by the ePWV equation, with long‐term all‐cause (primary outcome) and cardiovascular mortality (secondary outcome) in cancer survivors. A secondary aim was to identify an optimal ePWV threshold that best differentiates survival outcomes in cancer survivorship if a nonlinear association was observed. Our findings have the potential to inform tailored cardio‐oncology care and improve long‐term outcomes for cancer survivors at risk of CVD.

## METHODS

All data underlying this article are available at the National Health and Nutrition Examination Survey (NHANES) website at https://www.cdc.gov/nchs/nhanes/Default.aspx. The data that support the findings of this study are available from the corresponding author upon reasonable request.

### Data Sources

This analysis used data from the NHANES, which we restricted to cancer survivors. In this study, we define cancer survivors as all adult participants who self‐reported a diagnosis of cancer, regardless of treatment status or remission duration. The National Center for Health Statistics, part of the Centers for Disease Control and Prevention, produces this survey aimed at monitoring the health of the US population. The NHANES is a major program that generates a nationally representative sample of the civilian noninstitutionalized US population from about 5000 individuals each year in a 2‐year cycle. These are generated through a complex, multistage, probability‐sampling process, with oversampling of specific subgroups to improve the reliability and accuracy in these populations.^31^ Detailed sampling and data collection procedures have been previously published.^32^ In addition to demographic, socioeconomic, and health‐related questions, the surveys also include medical, physiological, and laboratory measurements.

### Study Design and Population

This was a retrospective cohort study that used 10 years of data from the 2009 to 2018 NHANES cycles. Data were linked with the NHANES Linked Mortality File, which links participants of NHANES aged ≥18 years with death records in the National Death Index data set through December 31, 2019, the latest mortality data available.^33^

### Study Sample

Survey responders aged ≥18 years, with or without CVD at baseline, who had both systolic and diastolic blood pressure data available, were included. Those who answered “yes” to the question, “Have you ever been told by a doctor or other health professional that you had cancer or a malignancy of any kind?” in the medical conditions questionnaire were identified as patients with cancer and included in this analysis. Those identified were further asked, “What kind of cancer?” and responses were recorded to specify the cancer type. The cancer types considered in our analysis included hematological (lymphoma and leukemia), bone, brain, breast, genitourinary (kidney, bladder, and prostate), gynecological (ovarian and uterine), gastrointestinal (esophageal, stomach, and colon), liver, gallbladder, pancreas, skin (melanoma, nonmelanoma, and others), soft tissue, and head and neck (thyroid, larynx, mouth, tongue, and lip) cancers.

We excluded cases ineligible for mortality follow‐up and those with incomplete data for variables, including hyperlipidemia, total cholesterol/high‐density lipoprotein cholesterol, body weight, and cancer type, resulting in the removal of 350 records from the initial sample; Figure S1 outlines the study flowchart.

### Baseline Characteristics, Exposure, and Measurements

Standardized questionnaires were used to collect baseline information on participants' demographic and clinical characteristics, including age, sex, race and ethnicity, education level, family income, smoking status, medical history, and medication use. Age was recorded as a continuous variable, while sex was categorized as male or female. Race and ethnicity were self‐reported in mutually exclusive categories: Mexican American, other Hispanic, non‐Hispanic White, non‐Hispanic Black, and other race. The household poverty index, a measure of socioeconomic status in NHANES, was calculated as the ratio of monthly family income to poverty levels and categorized into 4 groups: low (≤1.30), low‐middle (1.31–1.85), middle (1.86–3.50), and high income (>3.50). Education level was categorized as “less than high school,” “high school or equivalent,” or “more than high school.”

Participants self‐reported their medical history based on previous medical records from a health care professional or physician, including diabetes, hypertension, hyperlipidemia, coronary heart disease, congestive heart failure, heart attack, angina, and stroke. CVD was defined as the presence of any of the following conditions: congestive heart failure, coronary heart disease, angina, heart attack, or stroke. Family history of ischemic heart disease was also recorded. Medication use, including antihypertensive and lipid‐lowering medications, was defined by an affirmative response to whether a doctor or other health professional had recommended these treatments.

Blood pressure, body weight, and height were obtained from participants at NHANES examination centers following standardized protocols. Blood pressure was recorded using a standard sphygmomanometer after participants were seated quietly for 5 minutes. Measurements were taken from the right arm unless specific conditions precluded its use, or participants reported reasons for avoiding the right arm. Upper arm circumference was measured to ensure appropriate cuff size selection. Three consecutive blood pressure readings were obtained, and a fourth attempt was made if any reading was interrupted or incomplete. The average of at least 3 readings was used for analyses. Blood pressure measurement techniques adhered to the latest American Heart Association recommendations for human blood pressure measurement. Details about the quality assurance and quality control process are presented in the physician section of the Mobile Examination Center Operations Manual.^34^ Serum cholesterol (total, high‐density lipoprotein, and low‐density lipoprotein) levels were derived from venous blood samples collected after an 8‐hour fast, following a rigorous procedure outlined in the NHANES Procedures Manual for Laboratory/Medical Technologists.^34^

The primary exposure variable in this study was arterial stiffness estimated by ePWV, which was calculated using the following formula that first was described by Greve et al and derived by the Arterial Stiffness' Collaboration^35^: ePWV=9.587−(0.402×age)+[4.560×0.001×(age^2^)]−[2.621×0.00001×(age^2^×MBP)]+(3.176×0.001×age×MBP)−(1.832×0.01×MBP), where age is in years and mean blood pressure (MBP) is calculated by diastolic blood pressure +0.4×[systolic blood pressure—diastolic blood pressure].

### Study Outcomes

The primary outcome of interest in this study was all‐cause death, with a secondary outcome of cardiovascular death (*International Statistical Classification of Diseases*, *Tenth Revision* [*ICD‐10*] codes I00–I09, I11, I13, I20–I51, and I60–I69). Mortality status was assessed via a probabilistic record match to death certificate records from the National Death Index. Additional sources were used to determine mortality status, including those obtained via linkages with the US Social Security Administration or by active follow‐up of survey participants. Follow‐up time for each outcome was counted in months from the baseline examination date until the registered date of death or the end of the study (December 31, 2019), whichever occurred first.

### Ethical Approval

The NHANES data set research protocol was approved by the National Center for Health Statistics Ethics Review Board, and all participants provided informed consent. Since the data are publicly available, individual consent for this analysis was not required, and this research was exempt from institutional review board approval. This study adhered to the ethical principles for medical research as outlined in the Declaration of Helsinki.

### Statistical Analysis

All statistical analyses were conducted using R (R Foundation for Statistical Computing, Vienna, Austria) and SPSS version 28.0.0 (IBM, Armonk, NY). NHANES Mobile Examination Center weights were applied to account for oversampling, nonresponse, and noncoverage, ensuring nationally representative estimates. Combined weights for the 2009 to 2018 survey cycles were calculated and applied per NHANES guidelines, then rounded to the nearest integer. Details are available on the NHANES website (https://wwwn.cdc.gov/nchs/nhanes/tutorials/weighting.aspx). The normality of data distribution was assessed using the Shapiro–Wilk test. Continuous variables are reported as medians with interquartile ranges, while categorical variables are expressed as proportions. Comparisons between variables were conducted using the Pearson χ^2^ test or the Mann–Whitney *U* test, as appropriate.

Participants with high ePWV (≥9.4 m/s) were classified as having EVA, in line with previous large population‐based studies (38 population‐based cohorts in 11 European countries from the MORGAM [Monica Risk, Genetics, Archiving and Monograph] Project) that have used this threshold to define EVA on the basis of the upper quartile of ePWV in >100 000 apparently healthy individuals (aged 19–97 years).^36^ Survival distributions by EVA status were compared using log‐rank (Mantel–Cox) tests and visualized with Kaplan–Meier curves.

In the main analysis, Cox proportional hazard regression was used to assess the association between arterial stiffness estimated by the ePWV as a continuous variable (without any arbitrary risk thresholds) and the risk of all‐cause and cardiovascular death for the entire study cohort, followed by stratification across the following subgroups: EVA categories (yes/no), age (>60/<60 years), sex (male/female), known CVD (yes/no), and hypertension (yes/no). Three Cox models were fitted with stepwise adjustment: The crude model was unadjusted; model 1 adjusted for demographic variables and traditional cardiovascular risk factors not included in the ePWV equation, including age (>60/<60 years), sex, race, body mass index, hypertension, diabetes, smoking status, and hyperlipidemia; and model 2 further adjusted for socioeconomic factors (marital status, education level, and income), body and laboratory parameters (pulse rate, direct high‐density lipoprotein cholesterol, and total cholesterol), medication use (antihypertensive and antilipid medications), and cardiovascular comorbidities (congestive heart failure, heart attack, or stroke), to comprehensively account for potential confounding factors. These variables were selected on the basis of clinical relevance and prior literature. Follow‐up time, calculated from the date of examination to the date of death or the end of the study, was used as the underlying time measure for these models. Interaction terms were included to assess effect modification by age, sex, EVA status, CVD, and hypertension. None showed significant interaction (*P*>0.05). Results are presented as hazard ratios (HRs) with 95% CIs. All statistical analyses were 2‐tailed, and a *P* value <0.05 was considered statistically significant. As a supplementary analysis, we extended the fully adjusted model 2 from the main analysis by additionally adjusting for renal function, measured as estimated glomerular filtration rate (eGFR; mL/min per 1.73 m^2^), to consider the interplay between hemodynamic and renal dysfunction in risk stratification.

Proportional hazards assumptions for the Cox models were assessed using Schoenfeld residual testing.^37^ This method examines the correlation of time with the residuals between the observed and expected values of covariates at each failure time point. No significant correlation was found, indicating no proportional hazards violation. The variance inflation factor was also calculated to assess multicollinearity among the included variables in each model. No variables with a variance inflation factor ≥5 to 10 were found to be excluded.^38^

Restricted cubic spline analysis with 3 knots was used to assess the dose–response relationship between ePWV and outcomes across the entire study cohort. The *P* value for nonlinearity was calculated using the log‐likelihood ratio test. If a nonlinear association was observed, a sensitivity analysis was performed using the maximally selected rank statistics method to calculate a new ePWV cutoff value for groups. This complementary analysis, conducted via the “maxstat” package in R, determines the optimal ePWV threshold that best differentiates survival outcomes by testing all possible thresholds for the highest rank statistic.^39^ Participants were stratified on the basis of the new threshold, and survival outcomes reassessed using Kaplan–Meier curves and Cox models.

## RESULTS

A total of 105 869 670 weighted records (2234 unweighted) with a history of cancer were included in the analysis. All of them were adults, with a minimum age of 18 years, and had no missing vital status.

### Baseline Characteristics

The median age of the cancer population in our study was 65 years, with 44.2% being men. The racial distribution was predominantly non‐Hispanic White (86.4%), followed by non‐Hispanic Black (4.6%), Mexican American (2.7%), other Hispanic (2.6%), and other races (3.7%). The weighted median level of ePWV was 9.65 m/s. Participants had a median body mass index of 28.04 kg/m^2^ and a median pulse rate of 70 bpm. The median systolic blood pressure was 125.33 mm Hg, and the median diastolic blood pressure was 69.33 mm Hg. The prevalence of cardiovascular risk factors and comorbidities is summarized in (Table 1). The distribution of cancer types varied, with certain types showing higher representation (Table 1). All observed differences in Table 1 were statistically significant, with *P*<0.001.

**Table 1:** Survey‐Weighted Baseline Characteristics of the Study Cohort, Stratified by EVA Status (ePWV <9.4 m/s vs ≥9.4 m/s)

Characteristic	All cohort	Without EVA (low ePWV<9.4 m/s)	With EVA (high ePWV≥9.4 m/s)	*P* value
Records				
Unweighted, n	2234	825	1409	
Weighted, n (%)	105 869 670	48 012 155 (45.4)	57 857 515 (54.6)	
Estimated PWV, m/s, median (IQR)	9.65 (8.09–11.40)	7.87 (6.79–8.62)	11.23 (10.19–12.27)	<0.01
Age, y, median (IQR)	65 (54–75)	53 (44–59)	74 (68–80)	<0.01
Sex, %				
Men	44.2	38.7	48.7	<0.01
Women	55.8	61.3	51.3	
Race and ethnicity, %				
Mexican American	2.7	3.8	1.8	<0.01
Other Hispanic	2.6	3.7	1.6	
Non‐Hispanic White	86.4	83.2	89.1	
Non‐Hispanic Black	4.6	5.0	4.2	
Other race	3.7	4.3	3.3	
Education level, %				
Less than high school	10.6	9.0	11.8	<0.01
High school or equivalent	19.6	17.4	21.4	
More than high school	69.9	73.7	66.8	
Ratio of family income to poverty, %				
<1.31	14.0	16.0	12.4	<0.01
1.31–1.85	9.7	7.0	12.0	
1.86–3.5	25.9	21.1	30.0	
>3.5	50.3	55.9	45.7	
Marital status, %				
Married	62.6	64.5	61.0	<0.01
Widowed	13.5	3.5	21.7	
Divorced	12.6	13.5	11.8	
Separated	1.9	3.1	1.0	
Never married	5.8	9.4	2.8	
Living with partner	3.6	6.0	1.7	
Body mass index, kg/m^2^, median (IQR)	28.04 (24.50–33.00)	28.3 (24.5–33.8)	28 (24.4–32.1)	<0.01
Pulse rate, bpm, median (IQR)	70 (62–78)	76 (58–78)	72 (64–80)	<0.01
Blood pressure, mm Hg, median (IQR)				
Systolic blood pressure	125.33 (114.67–138.67)	116.67 (107.33–126.67)	132.67 (122.67–146.67)	<0.01
Diastolic blood pressure	69.33 (62.00–76.00)	70.00 (62.00–76.00)	68.67 (61.33–76.67)	<0.01
Mean blood pressure	87.56 (81.11–94.89)	85.11 (78.22–91.11)	90.22 (82.89–97.78)	<0.01
Direct HDL cholesterol, mmol/L, median (IQR)	1.34 (1.09–1.71)	1.29 (1.06–1.66)	1.37 (1.14–1.73)	<0.01
Total cholesterol, mmol/L, median (IQR)	4.94 (4.27–5.74)	4.99 (4.29–5.72)	4.91 (4.19–5.74)	<0.01
Smoking, %	15.3	23.9	8.1	<0.01
Diabetes, %	20.3	14.7	25.0	<0.01
Hypertension, %	51.7	38.9	62.3	<0.01
Antihypertensive medication(s), %	44.4	30.8	55.6	<0.01
Hyperlipidemia, %	53.3	45.0	60.1	<0.01
Antilipid medication(s), %	35.7	25.0	44.5	<0.01
Coronary heart disease, %	8.0	4.6	10.8	<0.01
Family history of IHD, %	15.5	18.3	13.2	<0.01
Congestive heart failure, %	5.6	2.9	7.8	<0.01
Heart attack, %	7.4	5.0	9.3	<0.01
Angina, %	4.6	4.3	4.8	<0.01
Stroke, %	6.1	3.9	8.0	<0.01
Number of cancers, %				
1	89.0	93.7	85.3	<0.01
2	9.8	5.9	12.9	
3	1.1	0.4	1.6	
>3	0.1	0.0	0.2	
Cancer type, %				
Uterus	3.3	3.9	2.8	<0.01
Thyroid	2.2	3.3	1.4	
Testis	1.3	2.6	0.3	
Stomach	0.3	0.6	0.1	
Skin (nonmelanoma)	31.5	16.6	31.8	
Rectum	0.3	0.1	0.6	
Prostate	9.6	9.8	4.2	
Pancreas	0.1	0.2	0.0	
Ovarian	1.8	2.2	1.4	
Mouth/tongue/lip	0.5	0.4	0.7	
Melanoma	8.2	7.6	8.7	
Lymphoma	1.5	2.2	0.9	
Lung	1.4	0.8	1.9	
Liver	0.5	0.4	0.6	
Leukemia	1.0	1.4	0.6	
Larynx	0.3	0.6	0.1	
Kidney	1.4	0.9	1.8	
Esophagus	0.3	20.7	0.1	
Colon	4.3	3.1	5.2	
Cervix	6.8	12.4	2.2	
Breast	14.7	52.9	12.5	
Brain	0.3	0.5	0.2	
Bone	0.2	0.3	0.2	
Blood	0.3	0.5	0.1	
Bladder	2.1	1.0	3.1	
	5.6	6.4	5.0	

ePWV indicates estimated pulse wave velocity; EVA, early vascular aging; HDL, high‐density lipoprotein; IHD, ischemic heart disease; IQR, interquartile range; and PWV, pulse wave velocity.

**Figure 1:**
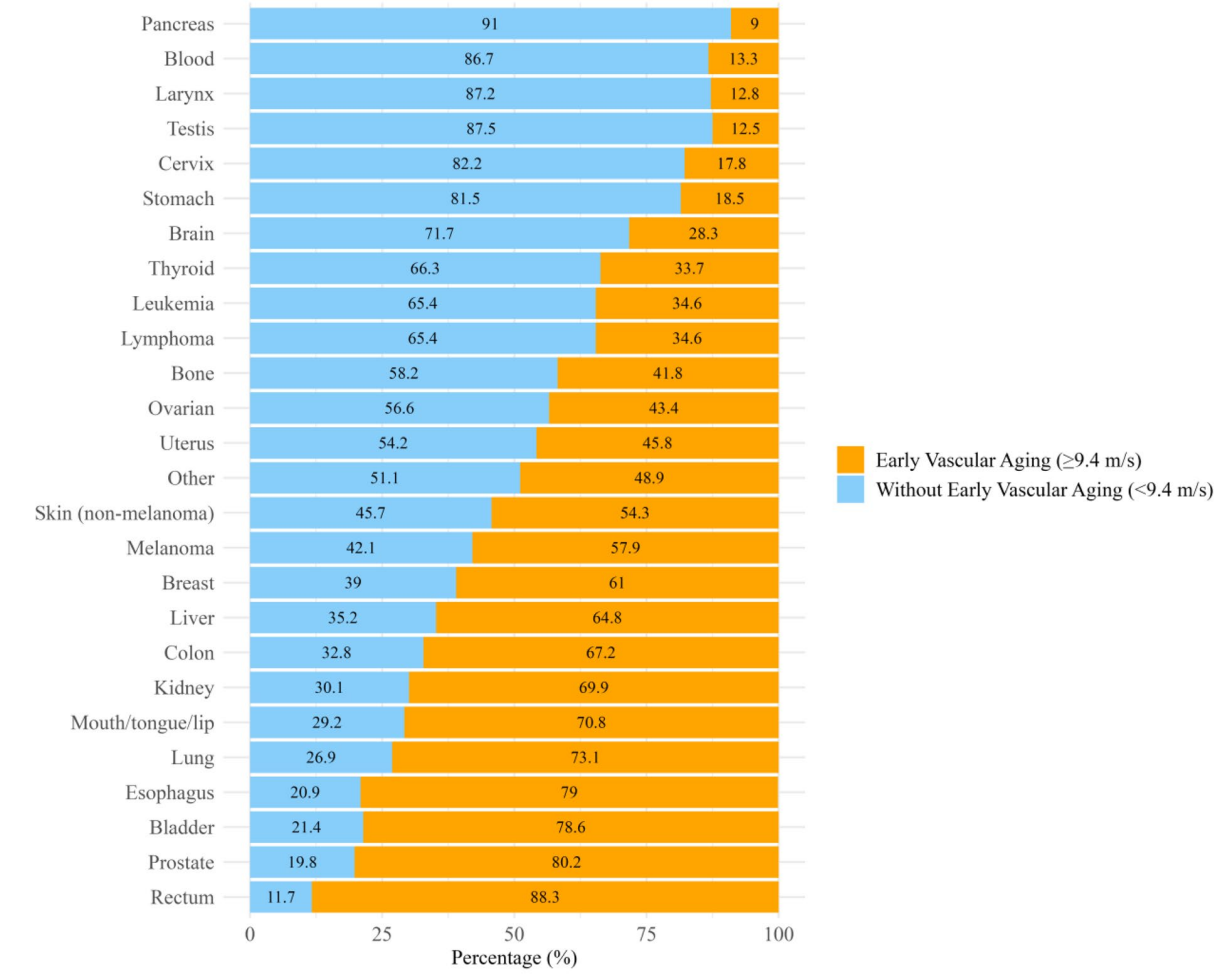
Distribution of early vascular aging (ePWV ≥9.4 m/s) among US adults with cancer, by cancer type: NHANES 2009 to 2018. ePWV indicates estimated pulse wave velocity; and NHANES, National Health and Nutrition Examination Survey.

Survivors of cancer with EVA (ePWV ≥9.4 m/s) comprised 54.6% of the cohort (Table 1). The distribution of demographics, comorbidities, cancer types, and cardiovascular risk factors between those with and without EVA showed significant differences across several characteristics, as outlined in (Table 1). Figure 1 illustrates the distribution of EVA across different cancer types. Survivors of rectal cancer have the highest proportion of EVA at 88.3%, followed by survivors of prostate cancer at 80.2%, esophageal (79%), and survivors of bladder cancer at 78.6%. Survivors of lung (73.1%) cancer also show high percentages of EVA. In contrast, survivors of pancreatic cancer have the lowest proportion of EVA at 9%, followed by survivors of testis (12.5%) and larynx (12.8%) cancer.

### Outcomes

During a median follow‐up period of 59 months (interquartile range, 33–91 months), 435 all‐cause and 91 cardiovascular deaths were recorded, equating to 14 979 486 weighted all‐cause deaths and 3 174 609 weighted cardiovascular deaths. Over up to 10 years of follow‐up, we observed a decrease in survival rates for those with EVA compared with those without.

**Figure 2:**
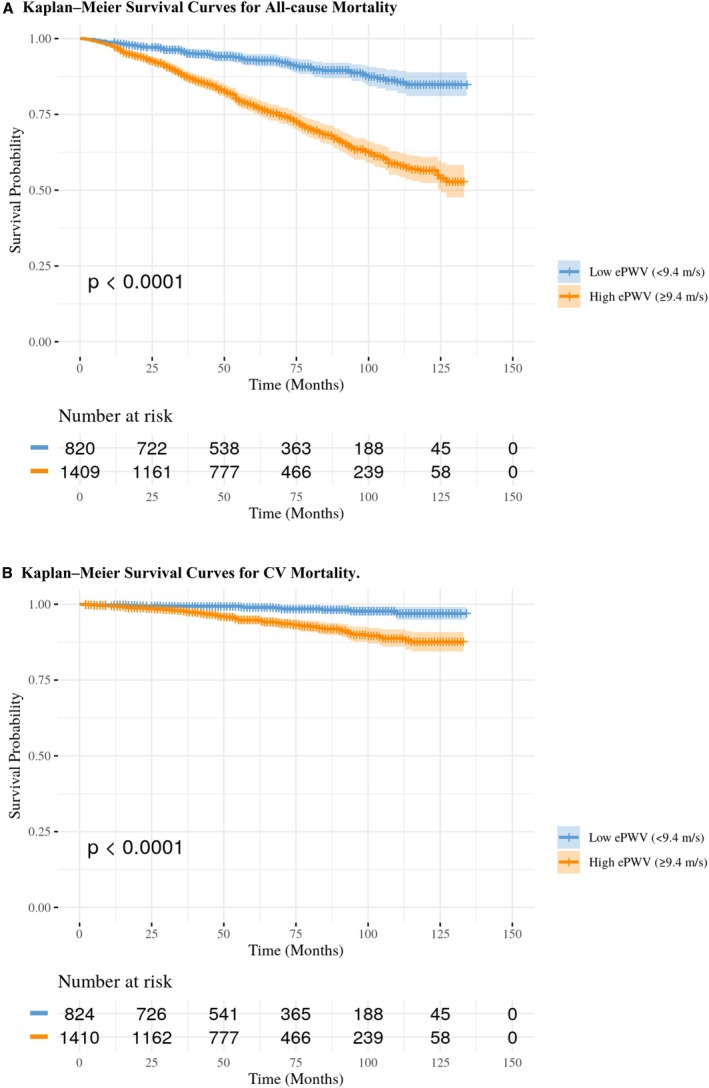
Kaplan–Meier survival curves by early vascular aging status (ePWV ≥9.4 m/s) in US adults with cancer (log‐rank *P*<0.001). **A,** Kaplan–Meier survival curves for all‐cause death and (**B**) Kaplan–Meier survival curves for cardiovascular death. CV indicates cardiovascular; and ePWV, estimated pulse wave velocity.

Figure 2 illustrates Kaplan–Meier analyses for all‐cause and cardiovascular death among survivors of cancer, stratified by EVA status. The plots show that survivors of cancer with EVA had significantly higher risks of both all‐cause death (Figure 2A) and cardiovascular death (Figure 2B) compared with those without EVA (*P*<0.001 by log‐rank test).

In an unadjusted Cox regression model, each 1‐m/s increase in ePWV was associated with a 40% increase in the risk of all‐cause death (HR, 1.40 [95% CI, 1.33–1.47]; *P*<0.001) and a 60% increase in the risk of cardiovascular death (HR, 1.60 [95% CI, 1.42–1.80]; *P*<0.01). After adjusting for demographic variables and traditional cardiovascular risk factors not included in the ePWV equation, including sex, age (>60/<60 years), race, body mass index, and comorbidities such as hypertension, diabetes, smoking, and hyperlipidemia (model 1), the associations remained significant (Table 2). In model 2, which included more comprehensive adjustments, covering marital status, education level, income, pulse rate, cholesterol levels, and cardiovascular comorbidities like congestive heart failure, heart attack, and stroke, the increased risk persisted. Specifically, each 1‐m/s increase in ePWV was linked to a 29% higher risk of all‐cause death (HR, 1.29 [95% CI, 1.19–1.40]; *P*<0.001) and a 46% higher risk of cardiovascular death (HR, 1.46 [95% CI, 1.20–1.76]; *P*<0.01). There were no significant interactions in these outcomes across racial groups, age, and known CVD status at the baseline, as all tested interaction terms had *P* values >0.05.

**Table 2:** Association Between ePWV (Continuous per 1 m/s) and Risk of All‐Cause and Cardiovascular Death

Outcome	Continuous ePWV per 1 m/s	
	HR (95% CI)	*P* value
All‐cause death* (n=435 [19.5%])		
Crude	1.40 (1.33–1.47)	<0.001
Model 1†	1.32 (1.23–1.42)	<0.001
Model 2†	1.29 (1.19–1.40)	<0.001
Cardiovascular death‡ (n=91 [4.1%])		
Crude	1.60 (1.42–1.80)	<0.01
Model 1†	1.48 (1.25–1.75)	<0.01
Model 2†	1.46 (1.20–1.76)	<0.01

ePWV indicates estimated pulse wave velocity; and HR, hazard ratio.

* *P* value for interactions=0.79 (age), 0.81 (sex), 0.67 (race), 0.18 (known cardiovascular disease).

^†^ Model 1 adjusted for sex, age >60/<60 y, race and ethnicity, body mass index, hypertension, diabetes, smoking history, and hyperlipidemia. Model 2 included adjustments for model 1 variables plus marital status, education level, income, pulse rate, direct high‐density lipoprotein cholesterol, total cholesterol, use of antihypertensive medication, use of antilipid medication, known cardiovascular disease(s); congestive heart failure, heart attack, and stroke.

^‡^ *P* value for interaction=0.27 (age), 0.07 (sex), 0.7 (race), 0.19 (known cardiovascular disease).

When the fully adjusted model was extended to account for renal function (eGFR, mL/min per 1.73 m^2^) in a subset of 17 127 487 weighted records (458 unweighted) with complete eGFR data, the association between ePWV and both all‐cause and cardiovascular death remained statistically significant (Table S1).

### Subgroup Analysis

**Figure 3:**
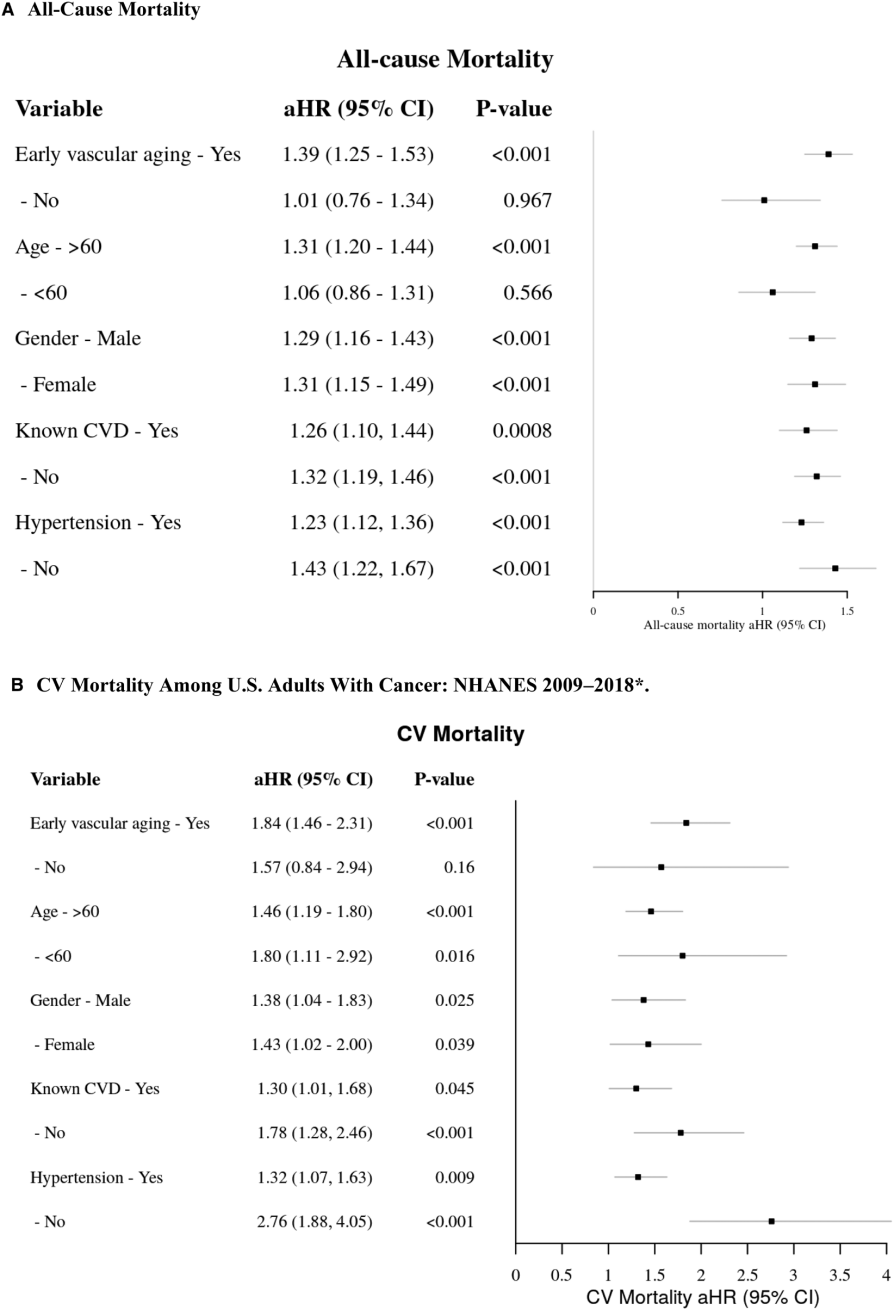
Subgroup analysis of the association between each 1‐m/s increase in ePWV and risk of (A) all‐cause death and (B) cardiovascular death among US adults with cancer: NHANES 2009 to 2018*. *Hazard ratios were adjusted for sex, age (>60/<60 y), race/ethnicity, body mass index, hypertension, diabetes, smoking history, and hyperlipidaemia, marital status, education level, income, pulse rate, direct high‐density lipoprotein cholesterol, total cholesterol, use of antihypertensive medication, use of antilipid medication, known cardiovascular disease(s), congestive heart failure, heart attack, and stroke. aHR indicates adjusted hazard ratio; CV, cardiovascular; ePWV, estimated pulse wave velocity; and NHANES, National Health and Nutrition Examination Survey.

Subgroup analyses were further conducted to assess the association between ePWV as a continuous variable and mortality outcomes, stratified by key clinical and demographic characteristics, including EVA status, age, sex, hypertension status, and the presence or absence of CVD (Figure 3). The Cox regression model, adjusted for all the aforementioned baseline characteristics and comorbidities, showed that for every 1‐m/s increase in ePWV, patients with EVA had a significantly higher risk of both all‐cause (HR, 1.39 [95% CI, 1.25–1.53]; *P*<0.001) and cardiovascular death (HR, 1.84 [95% CI, 1.46–2.31]; *P*<0.001) compared with those without EVA.

In individuals aged >60 years, an increase of 1 m/s in ePWV was strongly associated with increased all‐cause death (HR, 1.31 [95% CI, 1.20–1.44]; *P*<0.001) and cardiovascular death (HR, 1.46 [95% CI, 1.19–1.80]; *P*<0.001). Among those aged <60 years, the association with cardiovascular death remained significant (HR, 1.80 [95% CI, 1.11–2.92]; *P*=0.016). For men, each 1‐m/s increase in ePWV was associated with a significantly higher risk of all‐cause death (HR, 1.29 [95% CI, 1.16–1.43]; *P*<0.001) and cardiovascular death (HR, 1.38 [95% CI, 1.04–1.83]; *P*=0.025). Similarly, in women, each 1‐m/s increase in ePWV was significantly associated with an increased risk of all‐cause death (HR, 1.31 [95% CI, 1.15–1.49]; *P*<0.001) and cardiovascular death (HR, 1.43 [95% CI, 1.02–2.00]; *P*=0.039).

Among patients with preexisting CVD, each 1‐m/s increase in ePWV was associated with a significant increase in all‐cause death (HR, 1.26 [95% CI, 1.10–1.44]; *P*=0.0008) and cardiovascular death (HR, 1.30 [95% CI, 1.01–1.68]; *P*=0.045), and in those without CVD, the association of each 1‐m/s increase in ePWV with all‐cause death was stronger (HR, 1.32 [95% CI, 1.19–1.46]; *P*<0.001), as was the association with cardiovascular death (HR, 1.78 [95% CI, 1.28–2.46]; *P*<0.001). Similarly, in hypertensive individuals, each 1‐m/s increase in ePWV was associated with a significant increase in all‐cause death (HR, 1.23 [95% CI, 1.12–1.36]; *P*<0.001) and cardiovascular death (HR, 1.32 [95% CI, 1.07–1.63]; *P*=0.009), and in nonhypertensive individuals, the association of ePWV with outcomes was even more pronounced for all‐cause death (HR, 1.43 [95% CI, 1.22–1.67]; *P*<0.001), as was the association with cardiovascular death (HR, 2.76 [95% CI, 1.88–4.05]; *P*<0.001).

### Dose‐Dependent Relationship

**Figure 4:**
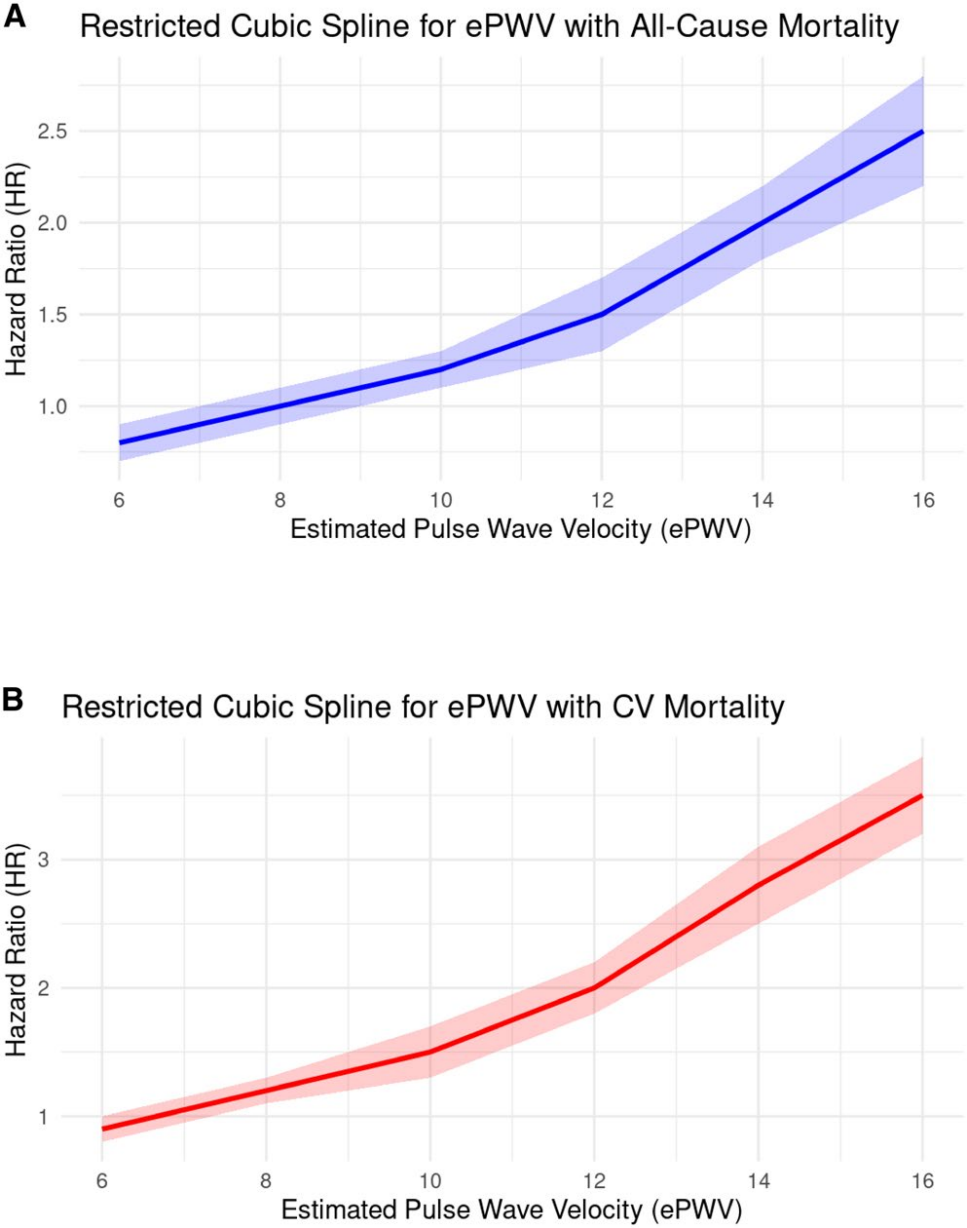
Restricted cubic spline curves for the dose–response relationship between ePWV and risk of (A) all‐cause death and (B) cardiovascular death among US cancer survivors: NHANES 2009 to 2018*. *Solid lines and transparent area represent HRs and 95% CIs, respectively. HRs (95% CI) were adjusted for the following variables: sex, age (>60/<60 y), race and ethnicity, body mass index, hypertension, diabetes, smoking history, and hyperlipidemia, marital status, education level, income, pulse rate, direct high‐density lipoprotein cholesterol, total cholesterol, use of antihypertensive medication, use of antilipid medication, known cardiovascular disease(s), congestive heart failure, heart attack, and stroke. CV indicates cardiovascular; ePWV, estimated pulse wave velocity; HR, hazard ratio; and NHANES, National Health and Nutrition Examination Survey.

Figure 4 illustrates a positive nonlinear relationship between ePWV and 10‐year mortality outcomes in survivors of cancer (*P* for nonlinearity<0.001). A steep rise in the risk of all‐cause death becomes visually evident when ePWV exceeds 12.5 m/s. Similarly, for cardiovascular death, a marked increase is also observed beyond 11.5 m/s. These findings highlight a stronger association between higher ePWV levels and elevated risks of all‐cause and cardiovascular death in the cohort of cancer survivors represented in this study.

### Sensitivity Analysis

Using the maximally selected rank statistics method, we identified 12.05 m/s as the optimal cutoff point for ePWV, allowing us to stratify survivors of cancer into 2 groups: ePWV <12.05 m/s versus ePWV ≥12.05 m/s. This threshold was selected as it provided the most effective separation in survival outcomes between the groups. Survey‐weighted differences in baseline characteristics between these groups are presented in Table S2.

In comparison with individuals with ePWV <12.05 m/s, those with ePWV ≥12.05 m/s demonstrated a significantly increased risk for both all‐cause and cardiovascular death (Table S3). These associations remained robust across 3 Cox models, with stepwise adjustments using the same covariates as in the main analysis. Figure S2 shows Kaplan–Meier analyses for all‐cause and cardiovascular death among survivors of cancer included in this sensitivity analysis, stratified by ePWV <12.05 m/s versus ePWV ≥12.05 m/s.

## DISCUSSION

This study aimed to investigate the association between arterial stiffness estimated by the ePWV equation, and long‐term all‐cause and cardiovascular death in survivors of cancer. Our findings demonstrate that each 1‐m/s increase in ePWV in survivors of cancer was significantly associated with a 29% higher risk of all‐cause death and a 46% higher risk of cardiovascular death at 10 years, after adjusting for demographic and socioeconomic variables, traditional cardiovascular risk factors not included in the ePWV equation, and other comorbidities.

The positive and nonlinear dose–response relationship between ePWV and mortality outcomes observed in our study cohort parallels findings from studies on the diabetic and stroke populations,^19^, ^24^ and highlights a disproportionate increase in mortality risk with higher ePWV thresholds. Survivors of cancer with ePWV exceeding 12.05 m/s exhibited significantly higher risks of mortality outcomes, with a 1.7‐fold increase in all‐cause death and a 2.5‐fold increase in cardiovascular death.

Although cfPWV remains the gold standard for assessing arterial stiffness, its use is limited in clinical practice due to the need for specialized equipment and expertise.^17^, ^18^, ^19^ ePWV, a simpler and more feasible alternative derived from age and MBP, has demonstrated a strong correlation with cfPWV and has been independently associated with death and cardiovascular events in the general population^18^, ^22^, ^27^, ^36^, ^40^ and other specific populations, including individuals with hypertension,^20^ diabetes,^19^ stroke,^14^ chronic kidney disease, and atherosclerotic CVD.^28^ Our study extends these observations to survivors of cancer, highlighting the value of ePWV as a prognostic tool for long‐term risk stratification in survivors of cancer with EVA. Beyond its prognostic significance, ePWV has also been associated with additional markers of arterial stiffness, including carotid stiffness, carotid intima‐media thickness, and carotid augmentation index, as demonstrated in the work of Heffernan et al.^22^ These associations further validate the utility of ePWV as a surrogate measure of arterial stiffness.

The concept of EVA aims to identify individuals whose vascular profile is disproportionately aged relative to their chronological age. Prior literature^41^ supports the utility of EVA in guiding cardiovascular risk stratification and clinical investigations, especially among individuals at increased cardiovascular risk. Survivors of rectal cancer showed the highest proportion of ePWV values in the range of EVA, followed by survivors of prostate, esophageal, and bladder cancer. In contrast, survivors of pancreatic, testicular, and laryngeal cancer had a lower prevalence of EVA. These disparities likely reflect differences in cancer treatment modalities, cardiovascular risk factor profiles, and cancer‐specific pathophysiology. Colorectal cancers share pathogenic mechanisms with atherosclerosis.^42^ Adjuvant chemotherapy used for colorectal cancers has been associated with increased arterial stiffness.^43^ In bladder cancer, treatment with androgen suppression also leads to an increased arterial stiffness.^44^ Similarly, alkylating agents such as cisplatin, commonly used in locally advanced or metastatic bladder cancers, impair vascular integrity and accelerate arterial stiffness.^45^

In survivors of cancer with EVA, survival rates over 10 years were significantly lower, accompanied by higher rates of hypertension (62.3% versus 38.9%) and hyperlipidemia (60.1% versus 45.0%, *P*<0.01), both of which are established contributors to arterial stiffness. Diabetes was also more prevalent in the EVA cohort (25.0% versus 14.7%, *P*<0.01), consistent with evidence linking diabetes to vascular remodeling via oxidative stress and extracellular matrix changes.^46^, ^47^ The EVA group had relatively higher MBP values (90.2 mm Hg versus 85.1 mm Hg). Smoking was notably less prevalent in the EVA group (8.1% versus 23.9%), potentially reflecting a more health‐conscious older population or survivorship bias. Use of antihypertensive (55.6% versus 30.8%) and lipid‐lowering medications (44.5% versus 25.0%) was also more common, likely indicating presence of longstanding cardiovascular risk or underlying vascular dysfunction. The early divergence of the Kaplan–Meier curves for all‐cause death (Figure 2A) around 12 months may be attributed, in part, to the age difference between the groups (73 versus 54 years and 62 versus 80 years, respectively), as advancing age is a well‐established determinant of arterial stiffness and cardiovascular risk. The interplay between age and arterial stiffness highlights the importance of age group–adjusted analyses to fully elucidate the independent contribution of EVA to long‐term outcomes in survivors of cancer.

Our subgroup analysis revealed that ePWV was an independent predictor of adverse outcomes in survivors of cancer, irrespective of age group, sex, hypertension status, or baseline CVD history. This supports the robustness of ePWV as a prognostic metric across diverse demographic and clinical profiles in cancer survivorship. Notably, ePWV demonstrated consistent associations with mortality outcomes across all age groups in our cohort, including those aged >60 years. In contrast, pulse pressure, traditionally used to assess arterial stiffness, showed limited prognostic value in older survivors of cancer,^30^ further indicating that pulse pressure does not provide substantial predictive information beyond systolic blood pressure.^48^

Arterial stiffness results from endothelial dysfunction, systemic inflammation, oxidative stress, and arterial wall remodeling.^6^ These processes increase left ventricular afterload, reduce coronary perfusion pressure, and contribute to myocardial oxygen imbalance, collectively promoting adverse cardiovascular outcomes.^49^, ^50^ In survivors of cancer, vascular toxicity from anticancer therapies compounds these risks and accelerates vascular aging. Anthracyclines, alkylating agents, and vascular endothelial growth factor inhibitors impair endothelial function, smooth muscle tone, and extracellular matrix integrity, thereby resulting in EVA.^45^ Cardio‐oncology guidelines currently emphasize structural changes in left ventricular function but often overlook the vascular changes that precede myocardial damage.^51^ Early monitoring of ePWV during and after cancer treatment may offer a valuable noninvasive tool for detecting arterial stiffness early, guiding targeted interventions, and potentially improving long‐term outcomes in cancer survivorship.

Strategies to address arterial stiffness include blood pressure control, lifestyle optimization, and pharmacological therapies, all aimed at mitigating the associated cardiovascular consequences. Effective management of blood pressure not only reduces systolic and pulse pressures but also decreases the left ventricular afterload, promoting efficient ventricular–vascular coupling.^52^ Lifestyle interventions, such as weight loss, regular moderate‐to‐vigorous physical activity, and smoking cessation, have demonstrated significant effects on slowing the progression of arterial stiffness by altering vascular wall remodeling and endothelial function.^53^, ^54^, ^55^ Pharmacological strategies include the use of angiotensin‐converting enzyme inhibitors, angiotensin receptor blockers, and mineralocorticoid receptor antagonists, which have shown pressure‐independent benefits in reducing arterial stiffness through their anti‐inflammatory and antifibrotic effects.^52^ Lipid‐lowering agents, particularly statins, may reduce arterial stiffness through pleiotropic effects, such as reducing oxidative stress and inflammation, although their impact appears modest.^56^ Moreover, novel antidiabetic therapies, including selective sodium–glucose cotransporter 2 inhibitors, such as empagliflozin, glucagon‐like peptide‐1 receptor agonists, and dipeptidyl peptidase‐4 inhibitors, have demonstrated potential in lowering arterial stiffness, particularly in individuals with type 2 diabetes.^57^

### Clinical Implications

Currently, there are no specific guidelines for monitoring vascular health with noninvasive and easy‐to‐measure markers in patients with cancer throughout treatment and survivorship. Our findings show that arterial stiffness, assessed by ePWV, offers a simple, noninvasive, and cost‐effective measure of vascular aging and helps to estimate long‐term risk of all‐cause and cardiovascular death in cardio‐oncology.

### Study Strengths and Limitations

This study is the first to assess ePWV's prognostic utility for all‐cause and cardiovascular mortality risk estimation in survivors of cancer using a large and nationally representative cohort. Mortality data were directly obtained from reliable nationwide registers, ensuring robust follow‐up. The study design has adjusted for a wide range of confounders. Our exploration of dose–response relationships and subgroup effects enhances the external validity of our findings. However, the study has limitations. The observational design precludes causal inference. Blood pressure measurements were performed only at baseline, and we lacked longitudinal data on blood pressure variability and changes in antihypertensive treatment. The absence of direct cfPWV measurements restricts comparisons with gold‐standard techniques. Self‐reported medical histories may introduce recall bias, and findings may not generalize to non‐US populations or specific cancer subtypes. While both eGFR and albumin‐to‐creatinine ratio are well‐established prognostic markers for cardiovascular outcomes in the general population,^58^ albumin‐to‐creatinine ratio could not be included in our analysis due to a high proportion of missing data across NHANES cycles. However, we conducted a supplementary analysis adjusting for eGFR in a subset of participants with complete data, which confirmed the robustness of the association between ePWV and death. We acknowledge the exclusion of albumin‐to‐creatinine ratio as a limitation and recommend that future studies incorporate both eGFR and albumin‐to‐creatinine ratio to better elucidate the interplay between haemodynamic and renal dysfunction in cardiovascular risk stratification frameworks for survivors of cancer. Moreover, we lacked data on cancer stage, grade, and treatment modalities, limiting the granularity of our analyses. Future studies should validate these findings across diverse cohorts and explore ePWV's integration into comprehensive cardio‐oncology models.

## CONCLUSIONS

Arterial stiffness, assessed by ePWV, demonstrates prognostic utility in survivors of cancer, showing a positive, nonlinear association with long‐term all‐cause and cardiovascular death, independent of demographic, socioeconomic, traditional cardiovascular risk factors not included in the ePWV equation, and baseline CVD status.

## Sources of Funding

None.

## Disclosures

None.

## Supporting information

Tables S1–S3
Figures S1–S2
