# Supplementary material for: Arterial Stiffness in Cancer Survivors: The Prognostic Value of Estimated Pulse Wave Velocity in US Cancer Population From the National Health and Nutrition Examination Survey 2009 to 2018
Source: J Am Heart Assoc. 2025 Sep 19;14(19):e041645. doi: 10.1161/JAHA.125.041645 (PMC12684599; doi:10.1161/JAHA.125.041645)
Supplement: Supplementary file 1 — Tables S1–S3 Figures S1–S2 [file JAH3-14-e041645-s001.pdf]

Supplemental Materials

Table S1: Association Between Estimated Pulse Wave Velocity (Continuous) and Risk of All-Cause and CV Mortality After Additional Adjustment for Renal Function (eGFR, mL/min/1.73 m²) in the Fully Adjusted Multivariable Model (Model 2) Across the Study Cohort with Available eGFR Data

Outcome	Continuous ePWV Hazard ratio per 1 m/s	
All-cause mortality	**HR (CI)**	**P value**
**Model 2***	1.24 (1.08 – 1.42)	0.002
CV mortality	**HR (CI)**	**P value**
**Model 2***	1.46 (1.04- 2.05)	0.025

*Model 2: adjusted for gender, age over/below 60, race/ethnicity, body mass index, hypertension, diabetes mellitus, smoking history, hyperlipidaemia, marital status, education level, income, pulse rate, **eGFR**, direct HDL cholesterol, total cholesterol, use of anti-hypertensive medication, use of anti-lipid medication, known cardiovascular disease(s); congestive heart failure, heart attack, and stroke. eGFR: estimated glomerular filtration rate. CV: cardiovascular. HR: hazards ratio, CI: confidence interval.

Table S2: Survey-Weighted Baseline Characteristics of Study Participants Stratified by Estimated Pulse Wave Velocity (ePWV <12.05 m/s vs ≥12.05 m/s)

**Characteristic**	**ePWV <12.05 m/s)**	**ePWV ≥12.05 m/s**	**P-value**
**Records, n**			
Unweighted	1,749	485	
Weighted	88,649,242	17,220,427	
**Estimated PWV (m/s),** median (IQR)	9.12 (7.79-10.45)	12.77 (12.40-13.22)	<0.01
**Age (years)**, median (IQR)	62 (52-70)	80 (79-80)	<0.01
**Gender, %**			<0.01
Men	44.1	44.4	
Women	55.9	55.6	
**Race/Ethnicity, %**			<0.01
Mexican American	3.0	1.1	
Other Hispanic	2.9	0.9	
Non-Hispanic White	85.2	92.6	
Non-Hispanic Black	4.7	3.7	
Other Race	4.2	1.6	
**Education level, %**			<0.01
Less than High School	9.3	17.0	
High school or equivalent	18.5	25.2	
More than High school	72.2	57.8	
**Ratio of family income to poverty, %**			<0.01
<1.31	14.4	12.3	
1.31-1.85	8.1	18.2	
1.86-3.5	24.0	36.4	
>3.5	53.5	33.0	
**Marital Status, %**			<0.01
Married	64.1	54.9	
Widowed	9.1	35.8	
Divorced	13.7	6.6	
Separated	2.3	0.3	
Never Married	6.5	2.0	
Living with partner	4.3	0.4	
**Body Mass Index (kg/m^2),** median (IQR)	28.42 (24.56-33.40)	26.85 (24.10-30.10)	<0.01
**Pulse rate**, median (IQR)	70 (64-78)	68 (62-76)	<0.01
**Blood pressure (mm Hg),** median (IQR)			
Systolic Blood Pressure	122.67 (112.00-132.67)	144.67 (134.67-156.00)	<0.01
Diastolic Blood Pressure	69.33 (62.00-76.67)	68.67 (63.33-75.33)	<0.01

Mean Arterial Pressure	86.44 (79.56-93.56)	94.22 (88.44-100.00)	<0.01
**Direct HDL-Cholesterol** (mmol/L), median (IQR)	1.32 (1.09-1.68)	1.42 (1.16-1.76)	<0.01
**Total Cholesterol** (mmol/L), median (IQR)	4.99 (4.27-5.77)	4.81 (4.06-5.53)	<0.01
**Smoking, %**	17.4	4.3	<0.01
**Diabetes Mellitus, %**	20.0	22.0	<0.01
**Hypertension, %**	48.2	70.0	<0.01
**Anti-hypertensive medication(s), %**	41.0	62.0	<0.01
**Hyperlipidaemia, %**	52.8	55.6	<0.01
**Anti-lipid medication(s), %**	34.2	43.1	<0.01
**Coronary heart disease**, %	7.0	13.3	<0.01
**Family history of IHD**, %	16.0	13.1	<0.01
**Congestive heart failure**, %	5.1	7.9	<0.01
**Heart attack**, %	7.0	9.3	<0.01
**Angina**, %	4.1	6.8	<0.01
**Stroke**, %	5.3	10.4	<0.01
**Number of cancers, %**			
1	90.1	83.4	
2	8.8	14.8	
3	1.0	1.5	
**Cancer type, %**			<0.01
Uterus	3.5	2.2	
Thyroid	2.6	0.5	
Testis	1.6	0.1	
Stomach	0.4	0.1	
Isolated non-melanoma skin cancers	31.6	30.9	
Rectum	0.4	0.3	
Prostate	8.8	13.6	
Pancreas	0.1	0	
Ovarian	1.9	1.0	
Mouth/tongue/lip	0.5	0.6	
Melanoma	8.5	6.6	
Lymphoma	1.5	1.4	
Lung	1.4	1.6	
Liver	0.6	0.1	
Leukemia	1.0	1.1	
Larynx	0.4	0	
Kidney	1.2	2.4	
Esophagus	0.2	0.6	
Colon	3.7	7.1	
Cervix	7.9	1.4	
Breast	14.0	18.1	
Brain	0.4	0.2	
Bone	0.2	0.3	
Blood	0.3	0	
Bladder	1.4	5.9	
Other	6.0	3.6	

IHD: Ischemic heart disease. ePWV: Estimated pulse wave velocity. IQR: Interquartile range. HDL: High-density lipoprotein. BMI: Body mass index. mm Hg: Millimeters of mercury

Table S3: Association Between Categorised Estimated Pulse Wave Velocity (ePWV) and Risk of All-Cause and CV Mortality

Outcome	ePWV (< **12.05 m/s)**	ePWV (≥**12.05) m/s)**	
All-cause mortality[1]	**Reference group**	**HR (CI)**	**P value**
Crude	NA	2.97 (2.45-3.61)	<0.001
**Model 1***	NA	1.88 (1.52-2.32)	<0.001
**Model 2***	NA	1.70 (1.35–2.13)	<0.001
CV mortality[2]	**Reference group**	**HR (CI)**	**P value**
Crude	NA	4.89 (3.24 – 7.39)	<0.01
**Model 1***	NA	2.84 (1.82 – 4.43)	<0.01
**Model 2***	NA	2.53 (1.56 – 4.12)	<0.01

*Model 1: adjusted for gender, age over/below 60, race/ethnicity, body mass index, hypertension, diabetes mellitus, smoking history, and hyperlipidaemia. Model 2: included adjustments for model 1 variables plus marital status, education level, income, pulse rate, direct HDL cholesterol, total cholesterol, use of anti-hypertensive medication, use of anti-lipid medication, known cardiovascular disease(s); congestive heart failure, heart attack, and stroke. [1]p-value for interaction: >0.05 for the following: age, gender, race, and known cardiovascular disease status. [2] p-value for interaction: > 0.05 for the following: age, gender, race, and known cardiovascular disease status. CV: cardiovascular. HR: hazards ratio, CI: confidence interval. ePWV: Estimated pulse wave velocity.

Figure S1: Study Flowchart

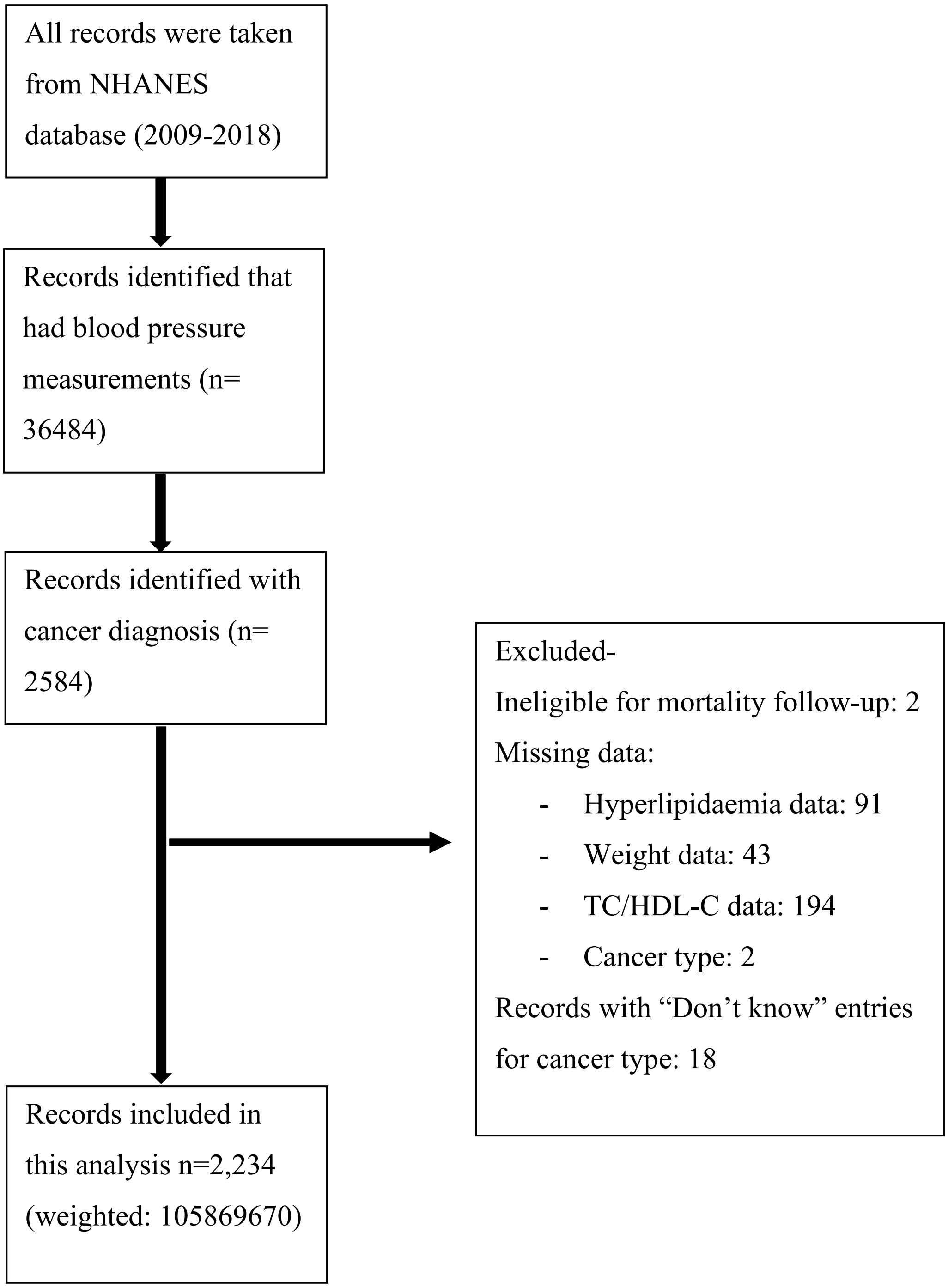

Figure S2: Kaplan–Meier Survival Curves for Categories of ePWV Defined by Optimal Cut-off Value Analysis (log-rank p-value < 0.001). a) Kaplan–Meier Survival Curves for All-cause mortality

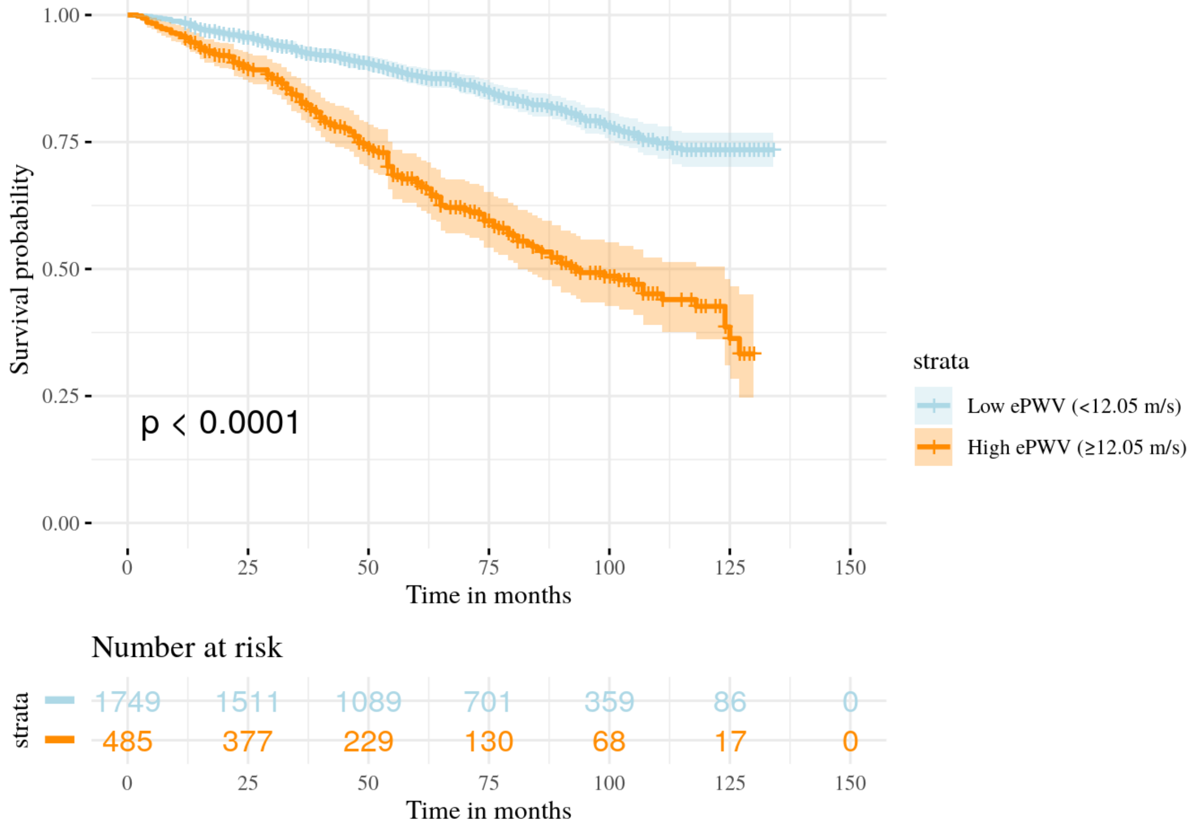

B) Kaplan–Meier Survival Curves for CV Mortality.

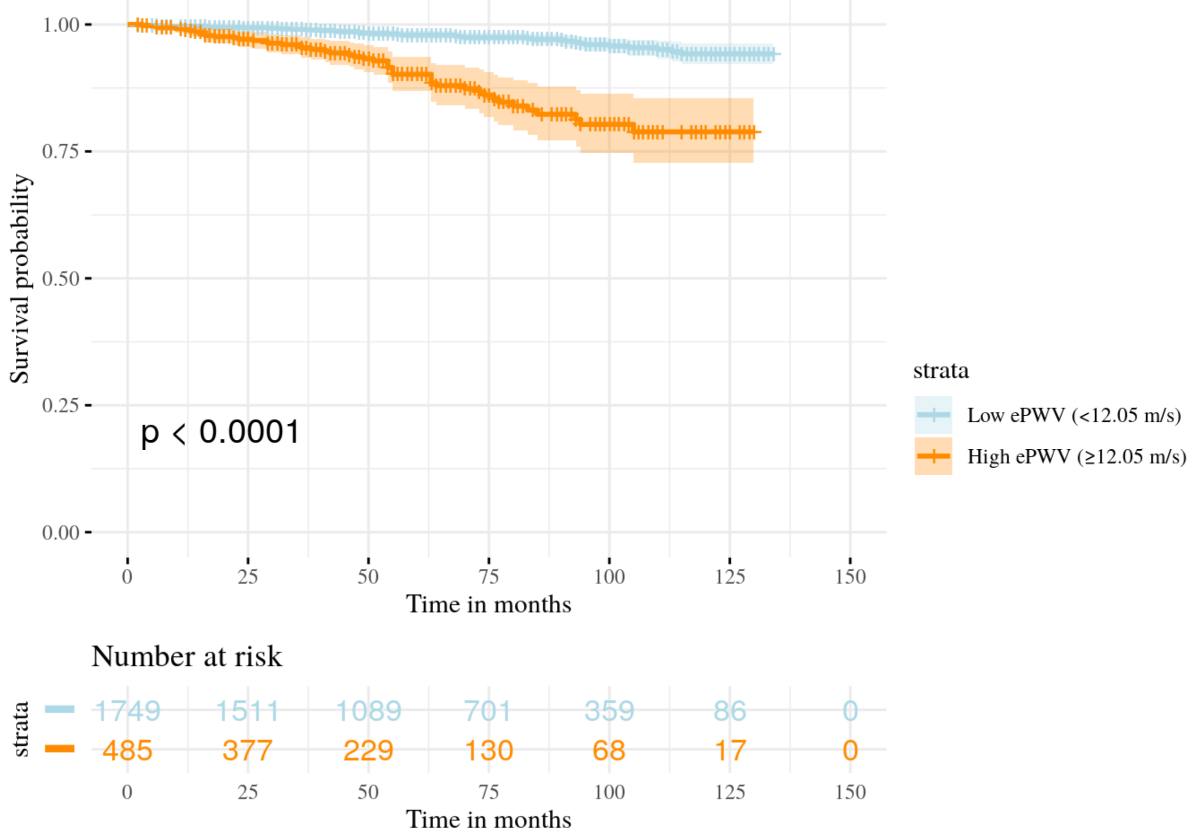

CV: cardiovascular. ePWV: Estimated pulse wave velocity.
